# Curcumol Targeting PAX8 Inhibits Ovarian Cancer Cell Migration and Invasion and Increases Chemotherapy Sensitivity of Niraparib

**DOI:** 10.1155/2022/3941630

**Published:** 2022-05-02

**Authors:** Caihong Liu

**Affiliations:** Clinical Medicine, The First Affiliated Hospital of Henan University of Science and Technology, Luoyang City, Henan Province 471003, China

## Abstract

**Objective:**

To investigate the effects of Curcumol on invasion, migration and epithelial-mesenchymal transformation of IGROV-1 and OVCAR-3 cells in ovarian cancer and its potential mechanism. Meanwhile, the effect of Curcumol on the antitumor activity of Niraparib was analyzed.

**Methods:**

Cell Counting Kit 8 (CCK-8) was used to detect the effects of Curcumol on the activity of IGROV-1 and OVCAR 3 cells. In vitro invasion assay (Transwell) was used to test the invasiveness of cells. Cell migration was detected by scratch assay. The inhibitory effect of Curcumol on PAX8 was detected by QRT-PCR. To evaluate the antitumor effect of Curcumol in subcutaneous tumor-bearing animal model.

**Results:**

Knockdown of PAX8 could inhibit the proliferation, invasion and migration of ovarian cancer cells. After Curcumol treated IGROV-1 and OVCAR-3 cells, the cell proliferation ability was decreased, the number of invasive cells was significantly reduced, and the scratch closure rate was significantly reduced, in a dose-dependent manner. Mechanism studies showed that Curcumol increased the antitumor activity of Niraparib by inhibiting PAX8.

**Conclusion:**

Curcumol can inhibit the invasion, migration and epithelial-mesenchymal transformation of IGROV-1 and OVCAR-3 cells in ovarian cancer, and its mechanism is related to the targeted inhibition of PAX8. Curcumol also increased the sensitivity of Niraparib chemotherapy by inhibiting PAX8.

## 1. Introduction

Ovarian malignant tumor is one of the three most common malignant tumors of female reproductive system, and its incidence is second only to cervical cancer and endometrial cancer [1]. Ovarian cancer is a gynecological malignant tumor with the highest mortality rate and the worst prognosis [2, 3]. The pathogenesis of ovarian cancer is still in the stage of research, and the exact mechanism has not been fully elucidated [4].

Curcumol is one of the most effective components of Curcumol oil, which has anti-tumor, anti-virus, anti-bacterial and anti-inflammatory effects [5]. Existing studies have shown that Curcumol has significant inhibitory effect on tumor cells and transplanted tumor mice in vitro [6]. The anticancer mechanism may be related to inhibiting tumor cell growth and RNA synthesis, enhancing specific immunity and producing obvious immune protection [7]. Studies have shown that Curcumol can affect cell membrane potential by killing tumor cells, inducing apoptosis and differentiation of tumor cells [8, 9]. Curcumol plays an anti-tumor role by inhibiting tumor nuclear metabolism, angiogenesis and anti-mutation [10]. It can inhibit the proliferation of cervical cancer, ovarian cancer, gastric cancer and nasopharyngeal cancer cells. However, the antitumor mechanism of Curcumol remains to be further studied.

Early diagnosis of ovarian cancer is particularly important for improving the prognosis of patients. The PAX gene family is ubiquitous in various animals [11]. It is highly conserved in evolution and can encode nuclear transcription factors [12]. Relevant studies have confirmed that this gene family is related to the normal development of organs and the occurrence and development of tumors [13]. PAX8 protein is encoded by the gene located on chromosome 2q12-14, with a molecular weight of 48kD and composed of 450 amino acids [14, 15]. It was first reported during thyroid development in mice. It was subsequently found to be highly expressed in follicular thyroid carcinoma. PAX8 plays a key role in the formation of thyroid, kidney, part of central nervous system, inner ear, eye, Wolffian canal and Mullerian canal organs during embryonic development [15, 16]. The fetus plays an important role in maintaining the normal function of cells after birth. Recent studies have found that PAX8 mutation may lead to the occurrence of thyroid cancer [17, 18]. At present, it has not been reported whether Curcumol can act on PAX8.

In this study, in vivo and in vitro experiments were conducted to analyze the effects of Curcumol on cell invasion, migration and epithelial mesenchymal transformation and its potential mechanism. Meanwhile, the effect of PAX8 on ovarian cancer cells was analyzed by cell assay. This study provides a basis for the subsequent study of Curcumol cancer inhibition and a potential target for the treatment of ovarian cancer.

## 2. Methods

### 2.1. Bioinformatics Analysis

The difference in PAX8 expression between the para-cancer control group and ovarian cancer tissues was analyzed by GEPIA (http://gepia.cancer-pku.cn/index.html). Through the human protein atlas (https://www.proteinatlas.org/) to obtain PAX8 in normal ovarian tissue and immunohistochemical staining results in ovarian cancer tissue.

### 2.2. Ovarian Cancer Tissue and Case Information

The specimens were collected from 16 patients who underwent surgery for malignant ovarian tumor in our hospital from February 2021 to August 2021.Tumor tissues and paracancer control tissues were collected. The patients were 39-71 years old, with an average age of (52.36 ± 6.93) years. Malignant tumors associated with genitalia and other systems were excluded. Reproductive tract infection, immune, endocrine and metabolic diseases were excluded. All patients underwent first surgery and did not receive hormone therapy before surgery. All specimens were collected with the consent of the patient and approved by the ethics Committee of The First Affiliated Hospital of Henan University of science and technology.

### 2.3. Cell Culture

The cells were inoculated in DMEM medium plus 10% FBS (Gibco, Life Technologies, Rockville, MD, USA). The culture condition is 37°C, 5%CO_2_ saturated moderate incubator (Thermo Fisher Scientific, Waltham, MA, USA). When the monolayer cell coverage rate was 80%, the cells at logarithmic growth stage were used for experiment.

### 2.4. Cell Transfection

The ovarian cancer cells were digested with trypsin and placed in a 6-well plate. The optimal cell density is 60% after 24 h culture. A mixture of shRNA and Lipofectamine 2000 (Life Technologies, Rockville, MD, USA) was prepared. ShRNA plasmids and their vectors are synthesized and supplied by GenePharma (Shanghai, China). Put them into the cell culture box and continue to culture for 48 h. Then they were used for subsequent cell proliferation experiments, fluorescence quantitative PCR experiments and other experiments.

### 2.5. Cell Proliferation Assay

The ovarian cancer cells were digested with trypsin, beaten, mixed and counted. The cell concentration was adjusted to 3 × 10^5^ cells/mL. Add to 3 96-well plates. 100 *μ*L/well, after laying the cells, culture for 72 h for detection. The detection procedure was as follows: add 10 *μ*L CCK-8 reagent into the well and incubate at 37°C for 2 ~ 3 h. The absorbance was measured at 450 nm by ELISA. After collating the data, statistical analysis was conducted.

### 2.6. Transwell Experiment

The substrate glue was diluted with serum-free medium and transferred to the upper compartment. Dry aseptic operating table. Cells at logarithmic growth stage were taken and the cell density was adjusted to 5 × 10^4^/mL in serum-free medium. Take 200 *μ*L cell suspension into the upper chamber, and add the corresponding concentration of Curcumol. Add 600 *μ*L medium containing 10% FBS to the lower chamber. After 24 hours of culture, the upper medium was discarded. Swab the cells in the upper layer and stain. Cells were counted and photographed in 3 fields under an inverted microscope.

### 2.7. Scratch Test

IGROV-1 and OVCAR-3 cells at logarithmic growth stage were taken and the cell density was adjusted to 1 × 10^6^/mL, and the cells were inoculated in 24-well plates. When cell coverage reaches 90%, draw parallel lines with 10 *μ*L of spear tip. PBS removed suspended cells and cell debris. Culture medium with different concentrations of Curcumol was changed for 24 h. Photos were taken at 0 h and 24 h. Scratch closure rate = (0 h scratch area -24 h scratch area)/(0 h scratch area) ×100%.

### 2.8. qRT-PCR

Collect the cells. The TRIzol method was used to extract total RNA. Use MMLV reverse transcription kit to reverse transcription of RNA into cDNA. Using this cDNA as a template, real-time fluorescence quantitative PCR detection kit was used to detect the expression of PAX8 in the above samples. The upstream primer sequence of PAX8 is 5' ~ AGGTGTGTGGAGAGAGAGATTTGG~3', and the downstream primer sequence is 5' ~ AGGTGAGGTTGAGTGGTTGC~3. The upstream primer sequence of GAPDH (internal reference) is 5' ~ CATGAGAGAGTATGAGACAGACAGCCCT~3', and the downstream primer sequence is 5' ~ AGTCCTTCCACGATACCAAGTT~3'. The primers were synthesized by Sangon Biotech (Shanghai) Co., Ltd. PCR reaction conditions: 95°C2min. 95°C for 10 s, 60°C for 30 s, a total of 40 cycles. The relative expression level of PAX8 was expressed as the value of 2^-*ΔΔ*Ct^, and the experiment was repeated 3 times.

### 2.9. Subcutaneous Graft Tumor Model

Cells at logarithmic growth stage were injected subcutaneously into the neck of nude mice (5 × 10^6^/100 *μ*L for each inoculated cell). A macroscopic tumor appeared at about 1 week. On the third day after tumor bearing, 12 nude mice were randomly divided into 2 groups according to body weight, namely model control group and Curcumol group, with 6 mice in each group. And begin medication treatment. Model control group was intraperitoneally injected with normal saline (0.2 mL/mouse). Curcumol group was intraperitoneally injected 100 mg/Kg. Intraperitoneal injection was given once every 3 days. Four weeks later, the tumor-bearing nude mice were sacrificed. The tumor tissue was completely exfoliated, the longest diameter and the shortest diameter of the tumor were measured, the tumor weight was weighed by electronic balance, and the tumor volume inhibition rate and tumor weight inhibition rate were calculated.

### 2.10. Statistical Analysis

SPSS 21.0 software was used for statistical analysis, and the measurement data (normal distribution) was expressed as (mean ± standard deviation). The t-test was used for pairwise comparison within the group. Pearson correlation analysis was adopted, and R stood for correlation coefficient. Comparisons between multiple groups were analyzed using one-way ANOVA. The statistical graph was drawn by GraphPadPrism7. P <0.01 was statistically significant.

## 3. Results

### 3.1. PAX8 Expression in Ovarian Cancer Tissues

**Figure 1:**
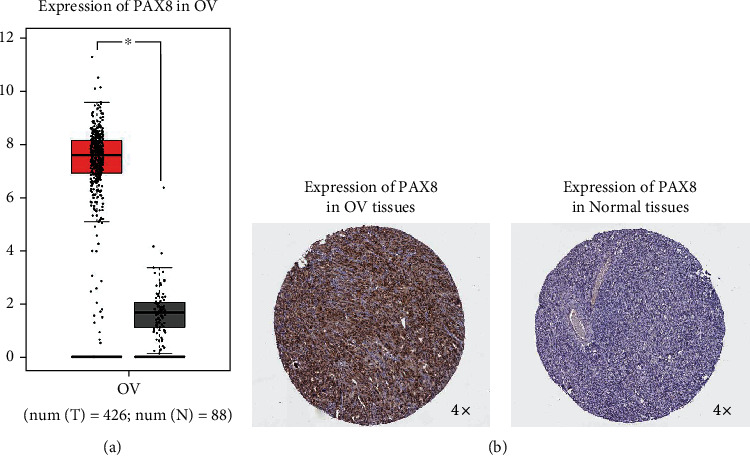
PAX8 is up-regulated in ovarian cancer. A. Bioinformatics analysis PAX8 is up-regulated in ovarian cancer. Data were obtained from GEPIA database (http://gepia.cancer-pku.cn/index.html).B. Immunohistochemistry verified that PAX8 is highly expressed in ovarian cancer. Data is obtained from the human protein atlas database (https://www.proteinatlas.org/).∗P <0.05,Tumor (n =426) vs Normal (n =88) tissues group.

PCR results showed that PAX8 was expressed in both ovarian cancer and para-cancer tissues, and the expression in ovarian cancer tissues was significantly higher than that in para-cancer control tissues (Figure 1(a)). That is, PAX8 is highly expressed in ovarian epithelial carcinoma tissues. Immunohistochemical results showed that PAX8 was expressed in both ovarian cysts and ovarian cancer tissues, and the expression in ovarian cancer tissues was significantly higher than that in the para-cancer control group (Figure 1(b)), that is, PAX8 was highly expressed in ovarian epithelial cancer tissues, which was consistent with the results of real-time quantitative PCR.

### 3.2. Correlation Analysis of THE Co-Expression of PAX8 and EMT Markers in Ovarian Epithelial Carcinoma

**Figure 2:**
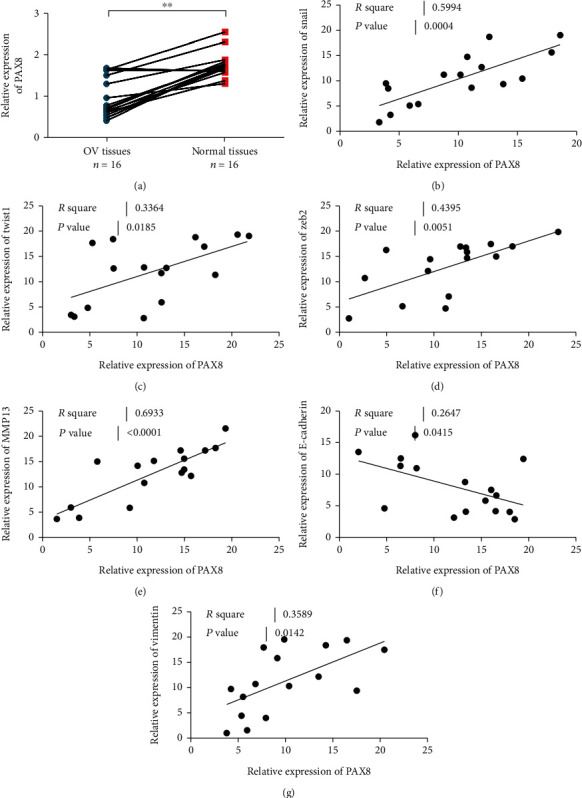
PAX8 is highly expressed in ovarian cancer. (a). The expression level of PAX8 in ovarian cancer tissues is higher than that in adjacent control tissues. N =16. (b). The expression level of PAX8 is positively correlated with the co-expression of Snail. (c). The expression level of PAX8 is positively correlated with the co-expression of Twsit1. (d). PAX8 expression is positively correlated with Zeb2 co-expression. (e). The expression of PAX8 is positively correlated with the co-expression of MMP13. (f). The expression of PAX8 is negatively correlated with the co-expression of E-cadherin. (g). PAX8 expression is positively correlated with Vimentin co-expression.

PCR results showed that PAX8 expression in ovarian cancer tissues was significantly higher than that in adjacent control tissues (Figure 2(a)). The correlation between PAX8 mRNA and transcription factors Snail, Twist1 and Zeb2 mRNA was analyzed. The results show that PAX8 is highly positively correlated with Snail, Twist1 and Zeb2 (Figures 2(b)-(D)). Correlation analysis was made between PAX8 and MMP13. The results showed that PAX8 was positively correlated with MMP13 (Figure 2(e)). Further detection showed that PAX8 was negatively correlated with epithelial marker e-cadherin (Figure 2(f)), while PAX8 was positively correlated with mesenchymal marker Vimentin (Figure 2(g)).

### 3.3. PAX8 Knockdown Inhibited EMT of Ovarian Cancer Cells

**Figure 3:**
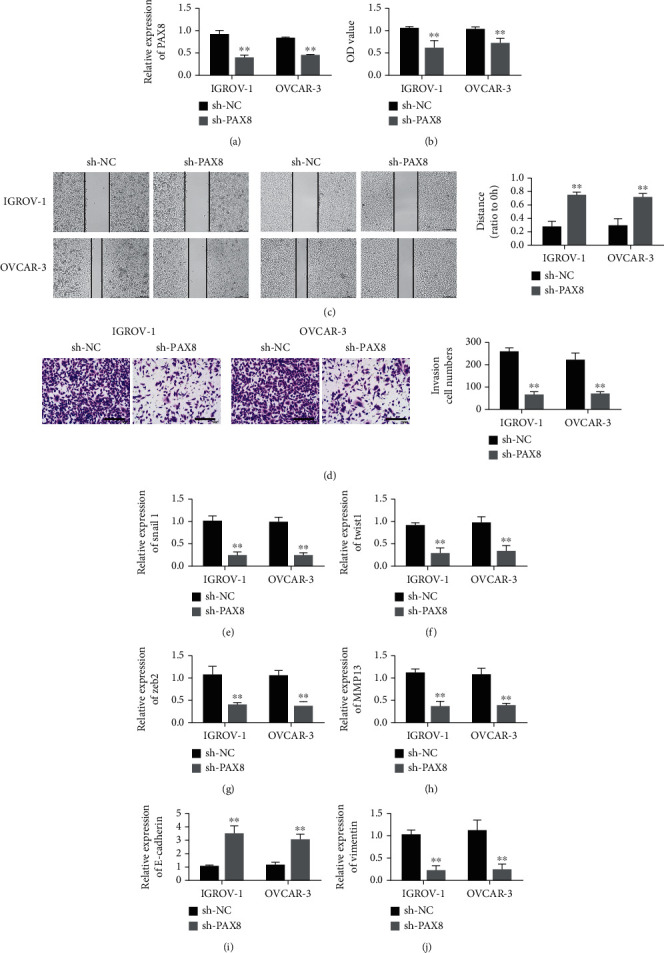
Knockdown of PAX8 inhibits the proliferation, invasion and EMT of ovarian cancer. (a). Verification of the efficiency of knocking down shRNA PAX8 in IGROV-1 and OVCAR-3 cells. (b). CCK-8 experiment verified that knocking down PAX8 inhibits the proliferation of ovarian cancer cells IGROV-1 and OVCAR-3. (c). Knockdown of PAX8 inhibits the migration ability of ovarian cancer cells IGROV-1 and OVCAR-3. (d). Knockdown of PAX8 inhibits the invasion of ovarian cancer cells IGROV-1 and OVCAR-3 by transwell assay. (e). After knocking down PAX8, detection of Snail1 expression in IGROV-1 and OVCAR-3 cells. (f). After knocking down PAX8, detection of Twist1 expression in IGROV-1 and OVCAR-3 cells. (g). Detection of Zeb2 expression in IGROV-1 and OVCAR-3 cells after knocking down PAX8. (h). After knocking down PAX8, detection of MMP13 expression in IGROV-1 and OVCAR-3 cells. (i). After knocking down PAX8, detection of E-cadherin expression in IGROV-1 and OVCAR-3 cells. (j). After knocking down PAX8, detection of Vimentin expression in IGROV-1 and OVCAR-3 cells. ∗P <0.05, ∗∗P <0.01, vs sh-NC group.

PAX8 mRNA level was detected by real-time quantitative PCR. The results showed that the PAX8 gene expression level of ovarian cancer cells was decreased after transfection with low knockdown plasmid compared with the control group, and the difference was statistically significant (Figure 3(a)). CCK8 results showed that compared with the control group, cell proliferation activity was inhibited after PAX8 gene interference, with statistically significant difference (Figure 3(b)). Cell scratch and Transwell results showed that cell migration and invasion were inhibited after PAX8 gene interference compared to the control group (Figures 3(C)-(D)). Snail, Twist1, Zeb2, MMP13 in Figures 3(e)-(h). Compared with the control group, Curcumol up-regulated the expression of e-cadherin in IGROV-1 and OVCAR 3 cells, and decreased the expression of Vimentin in IGROV-1 and OVCAR 3 cells (Figures 3(I)-(j)). Curcumol can inhibit the EMT process of ovarian cancer cells.

### 3.4. Curcumol Inhibits Ovarian Cancer Cell Proliferation and Invasion by Inhibiting PAX8 Expression

**Figure 4:**
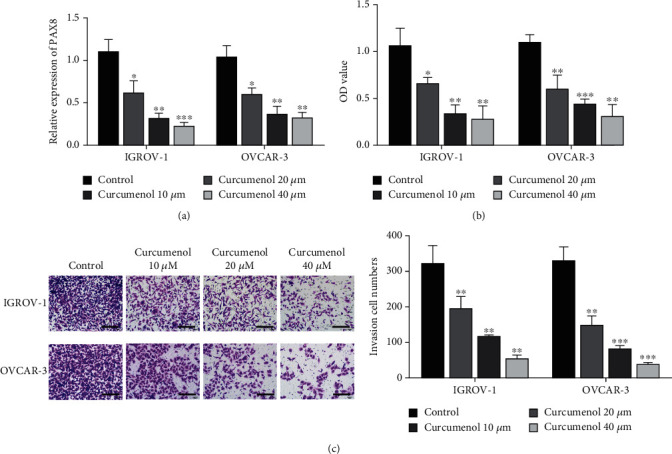
Curcumol inhibits the expression of PAX8 and inhibits the malignant progression of ovarian cancer cells. (a). Detection of PAX8 expression in IGROV-1 and OVCAR-3 cells after treatment with different concentrations of Curcumol (10, 20, 40 *μ*M). (b). After treatment with different concentrations of Curcumol, the proliferation ability of IGROV-1 and OVCAR-3 cells was detected by CCK-8. (c). After treatment with different concentrations of Curcumol, the invasion ability of IGROV-1 and OVCAR-3 cells was detected by Transwell assay. ∗P <0.05, ∗∗P <0.01, ∗∗∗P <0.001, vs Control group.

Curcumol could inhibit the expression of PAX8 in IGROV-1 and OVCAR-3 cells in a dose-dependent manner (Figure 4(a)). CCK-8 assay was used to detect the effects of Curcumol on IGROV-1 and OVCAR 3 cell viability. The results showed that Curcumol inhibited IGROV-1 and OVCAR-3 cells significantly after treated with IGROV-1 and OVCAR-3 cells for 48 h (Figure 4(b)). Transwell results showed that different concentrations of Curcumol treated IGROV-1 and OVCAR-3 cells significantly reduced the number of invading cells in single field. Statistical results also showed that the number of invasive cells was significantly reduced after Curcumol treatment of IGROV-1 and OVCAR-3 cells, and the effect had a Curcumol dose-dependent effect (Figure 4(C)).

### 3.5. Curcumol Inhibits Ovarian Cancer Tumor Proliferation by Inhibiting PAX8 Expression

**Figure 5:**
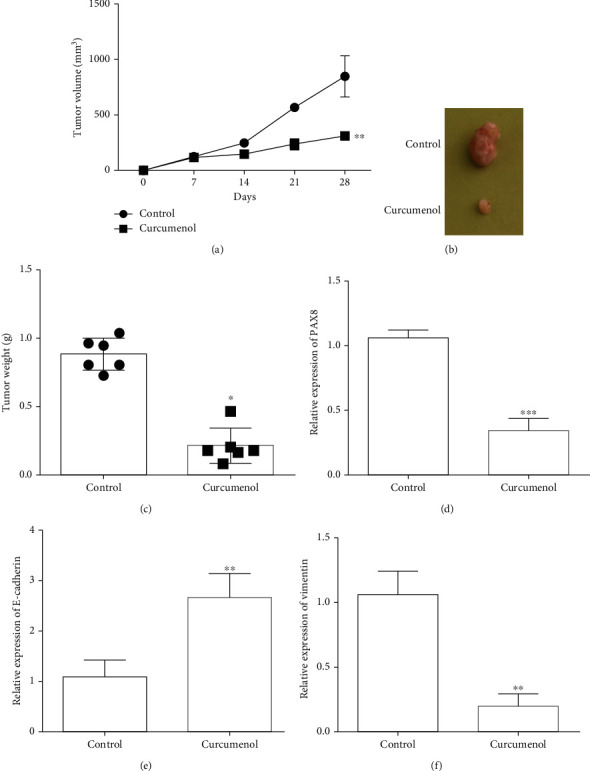
Subcutaneous tumor-bearing experiments in nude mice prove that Curcumol inhibits the proliferation and metastasis of ovarian cancer cells. (a). Tumor growth curve of nude mice (Curcumol: 100 mg/kg). (b). Gross picture of nude mouse tumor. (c). Tumor weight of nude mice. (d). Detection of the expression level of PAX8 in two groups of tumor tissues. (e). The expression of E-cadherin in the control and Curcumol treated tissue was detected. (f). Vimentin expression in the control and Curcumol treated tissue was detected. N =6. ∗P <0.05, ∗∗P <0.01, ∗∗∗P <0.001, vs Control group.

After Curcumol treated subcutaneous animal model of ovarian cancer cell line, tumor proliferation rate decreased (Figure 5(a)). Tumor volume detection results showed that compared with the control group, the subcutaneous tumor volume and tumor weight of nude mice were decreased after Curcumol treatment (Figures 5(b)-(C)). The expression of PAX8 was further detected by QRT-PCR. Experimental results showed that the expression level of PAX8 in tumor tissues was significantly decreased after Curcumol treatment (Figure 5(D)). Compared with the control group, Curcumol up-regulated the expression of e-cadherin and decreased the expression of Vimentin in tumor tissues (Figures 5(e)-(f)). Curcumol can inhibit the EMT process of ovarian cancer cells.

### 3.6. Knockdown of PAX8 or Combination with Curcumol Can Increase Chemotherapy Sensitivity of Ovarian Cancer

**Figure 6:**
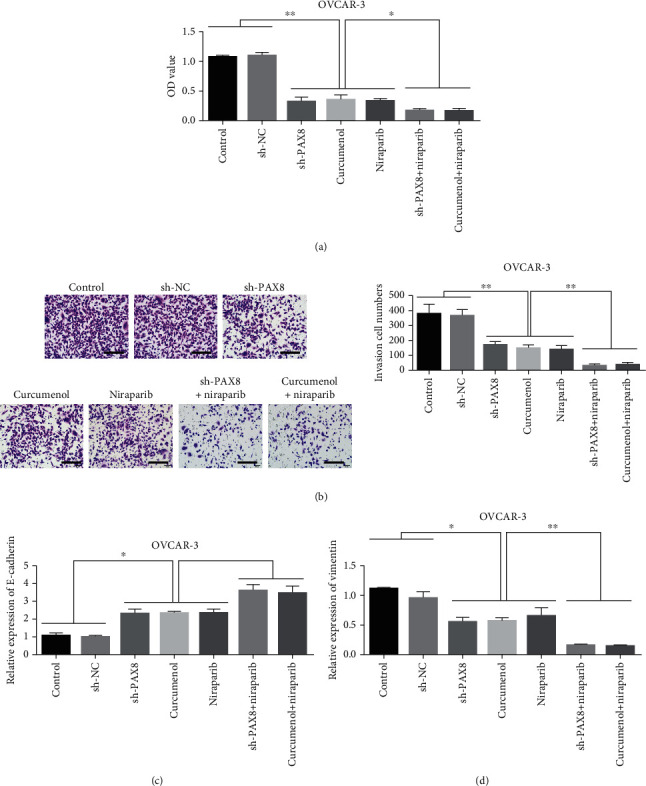
Knockdown of PAX8 or combination of Curcumol can increase the sensitivity of ovarian cancer chemotherapy. (a). After different treatments, cell proliferation detection. (b). After different treatments, cell Transwell detection. (c). After treatment with different concentrations of Curcumol, the expression of E-cadherin in OVCAR-3 cells was detected. (d). Detection of Vimentin expression in OVCAR-3 cells after treatment with different concentrations of Curcumol. ∗P <0.05, ∗∗P <0.01.

CCK-8 detected the changes of cell proliferation ability after different treatments. Knockdown of PAX8 or Curcumol reduced OVCAR 3 proliferation compared with control. The cell proliferation capacity was the lowest after simultaneous use of Niraparib (Figure 6(a)). Transwell detected changes in the invasiveness of cells after different treatments. The results showed that both PAX8 knockdown and Curcumol treatment reduced OVCAR 3 invasiveness compared with control. Meanwhile, the combination of Niraparib showed the lowest cell invasion ability (Figure 6(b)). Qrt-pcr results showed that knockdown of PAX8 or combined use of Curcumol could increase the expression of E-cadherin (Figure 6(c)). While PAX8 knockdown or Curcumol combination can increase the expression of Vimentin inhibition (Figure 6(d)).

## 4. Discussion

Ovarian cancer is a malignant tumor with high mortality rate of female reproductive system [19]. Although great progress has been made in clinical diagnosis and treatment of ovarian cancer in recent years, the survival rate of patients is still relatively low [20].

In recent years, TCM monomer extracts have made great progress in cancer treatment and adjuvant therapy [21–24]. For example, studies on monomers such as ginsenoside and astragaloside IV have entered clinical stage [25, 26]. Curcumol is a monomer compound extracted from Zedoary turmeric, which has an inhibitory effect on nasopharyngeal cancer, gastric cancer, colon cancer, bile duct cancer and other cancer cells [27]. Chen and Hua [28] found that Curcumol combined with celecoxib can enhance its inhibitory effect on lung cancer migration by inhibiting the activation of PI3KAKT pathway. In breast cancer cells, Curcumol inhibits cell metastasis by inhibiting the expression of NF-*κ*B pathway dependent matrix metalloproteinase 9 [29]. Qi et al. found that eEF1A1 was a molecular target of Curcumol and was involved in inhibiting the metastasis of breast cancer MDA-MB-231 cells through proteomics methods [30]. Wang reported that Curcumol coated liposome nanocomplexes showed an inhibitory effect on ovarian cancer [31]. Results of TANG and Han et al. showed that Curcumol could inhibit the proliferation and migration of human ovarian cancer cell line SKOV3 by inhibiting JAK2/STAT3 signaling pathway [32, 33]. The results of this study were similar to those of the above studies. After Curcumol treatment of ovarian cancer cells, the number of invasive cells was significantly reduced, and the degree of scratch closure was significantly reduced. The results showed that Curcumol inhibited the invasion and migration of ovarian cancer cells.

With the deepening of the research on the mechanism of drug resistance of ovarian cancer cells, VEGF, NANOG, epithelial cadherin, Snail, etc., have been found to play an important role in drug resistance of ovarian cancer, and are expected to become new targets for the treatment of ovarian cancer [34]. Currently, drugs targeting molecular targets have been proven to reverse tumor drug resistance. Yang et al. [35] found that cd44-targeting HA-PEI/HA-PEG/MDR1 small interfering RNA nanoparticles can be used as a tool to reverse multidrug resistance of tumor cells. The multi-kinase inhibitor BML-275 and its analogue LDN-193189 were found to be able to re-sensitize drug-resistant ovarian cancer cells to the killing effect of platinum drugs by inducing autophagy and apoptosis, respectively, which is expected to become a new drug for the treatment of ovarian cancer [36]. In addition, studies have found that patients with CHD4 deletion are sensitive to chemotherapy, but the deletion of CHD4 in BRCA2 mutant cells can enhance the resistance of cancer cells to cisplatin and PARP inhibitors by opening a channel known as “DNA damage tolerance” [37].

PAX8 belongs to the transcription factor pairing box gene family and is a cell line limiting transcription factor [38]. The PAX family proteins are highly conserved regions formed by 128 amino acids, including PAXL-9. The matching box can identify specific DNA binding sites, initiate transcription, and control gene regulation [39]. This study showed that the expression of PAX8 in ovarian cancer tissues was significantly higher than that in adjacent tissues. PAX8 is highly correlated with EMT-related transcription factors. These results suggest that PAX8 may be involved in the development and progression of ovarian cancer. This study provides a theoretical basis for exploring the pathogenesis of ovarian cancer inflammation, and also provides guidance for the prevention and treatment of ovarian cancer. PAX8 is a regulator of embryonic tissue development and cell differentiation, and is highly expressed in epithelial ovarian cancer tissues [40, 41]. It has been reported that PAX8 controls the cell cycle and metabolic gene expression program in ovarian cancer cells by binding to enhancers [42, 43]. In this study, Curcumol inhibited PAX8 expression in ovarian cancer cells. These results suggest that Curcumol may play an inhibitory role in cancer through PAX8.

Niraparib is a novel PARP inhibitor and the first targeted drug for recurrent ovarian epithelial carcinoma [44]. Niraparib has good therapeutic effect on patients with wild-type and mutant types of breast Cancer susceptibility gene (BRCA) [45]. In this study, Curcumol increased the chemotherapeutic sensitivity of Niraparib to ovarian cancer.

## 5. Conclusion

In this study, Curcumol inhibited the proliferation, invasion and migration of ovarian cancer cells. In vivo results showed that Curcumol inhibited the growth of ovarian cancer transplanted from nude mice and had synergistic effect with Niraparib, which may be related to the down-regulation of PAX8 expression. This study provides a basis for the application of Curcumol ovarian cancer, but it needs to be confirmed by further molecular and clinical experiments.

## Data Availability

The analysed data sets generated during the study are available from the corresponding author on reasonable request.
